# Mapping and Analysis of the Connectome of Sympathetic Premotor Neurons in the Rostral Ventrolateral Medulla of the Rat Using a Volumetric Brain Atlas

**DOI:** 10.3389/fncir.2017.00009

**Published:** 2017-03-01

**Authors:** Bowen Dempsey, Sheng Le, Anita Turner, Phil Bokiniec, Radhika Ramadas, Jan G. Bjaalie, Clement Menuet, Rachael Neve, Andrew M. Allen, Ann K. Goodchild, Simon McMullan

**Affiliations:** ^1^Faculty of Medicine and Health Sciences, Neurobiology of Vital Systems, Macquarie UniversitySydney, NSW, Australia; ^2^Department of Anatomy, Institute of Basic Medical Sciences, University of OsloOslo, Norway; ^3^Department of Physiology, University of MelbourneMelbourne, VIC, Australia; ^4^Viral Core Facility, McGovern Institute for Brain Research, Massachusetts Institute of TechnologyCambridge, MA, USA

**Keywords:** connectome, RVLM, sympathetic, rabies, volumetric, segmentation, respiratory-sympathetic, mesoscale

## Abstract

Spinally projecting neurons in the rostral ventrolateral medulla (RVLM) play a critical role in the generation of vasomotor sympathetic tone and are thought to receive convergent input from neurons at every level of the neuraxis; the factors that determine their ongoing activity remain unresolved. In this study we use a genetically restricted viral tracing strategy to definitively map their spatially diffuse connectome. We infected bulbospinal RVLM neurons with a recombinant rabies variant that drives reporter expression in monosynaptically connected input neurons and mapped their distribution using an MRI-based volumetric atlas and a novel image alignment and visualization tool that efficiently translates the positions of neurons captured in conventional photomicrographs to Cartesian coordinates. We identified prominent inputs from well-established neurohumoral and viscero-sympathetic sensory actuators, medullary autonomic and respiratory subnuclei, and supramedullary autonomic nuclei. The majority of inputs lay within the brainstem (88–94%), and included putative respiratory neurons in the pre-Bötzinger Complex and post-inspiratory complex that are therefore likely to underlie respiratory-sympathetic coupling. We also discovered a substantial and previously unrecognized input from the region immediately ventral to nucleus prepositus hypoglossi. In contrast, RVLM sympathetic premotor neurons were only sparsely innervated by suprapontine structures including the paraventricular nucleus, lateral hypothalamus, periaqueductal gray, and superior colliculus, and we found almost no evidence of direct inputs from the cortex or amygdala. Our approach can be used to quantify, standardize and share complete neuroanatomical datasets, and therefore provides researchers with a platform for presentation, analysis and independent reanalysis of connectomic data.

## Introduction

The nascent field of *connectomics* applies rapidly developing ultrastructural, trans-synaptic tracing, and whole brain imaging technologies to identify neural circuits at micro-, meso-, and macroscopic resolutions. The central tenet of the connectomic approach is that insights regarding both the functional properties of specific neural circuits and general brain organizational principles may be gained by definitively resolving network architecture (Carandini, 2012; Denk et al., 2012; Mitra, 2014).

Ultrastructural approaches such as serial block-face electron microscopy can comprehensively identify local synaptic connectivity, but are limited by the enormous time and costs associated with data acquisition and analysis, and are therefore best suited to the examination of small regions of brain in high detail (discussed by Lichtman and Denk, 2011; Wanner et al., 2015). Investigators interested in mapping more diffuse circuits have instead monitored the trans-synaptic spread of replication-competent neurotropic viruses such as rabies (Ugolini, 1995; Kelly and Strick, 2000; Dum et al., 2016) and alpha herpes variants (Strack et al., 1989; Rinaman and Schwartz, 2004; McGovern et al., 2012; reviewed by Nassi et al., 2015; Wojaczynski et al., 2015).

In recent years glycoprotein-deleted EnvA-pseudotyped rabies [SADΔG(EnvA)] has emerged as a flagship tool for tracing connectomes in experimental animals (Wickersham et al., 2007b; Callaway and Luo, 2015). The key insights made by Callaway and colleagues in developing this system are that the ability of SADΔG(EnvA) to enter populations of target neurons and retrogradely spread to monosynaptically connected partners can be controlled by selective expression of the EnvA receptor, TVA, and the rabies glycoprotein respectively. Here we apply this approach to resolve the afferent connectome of putative sympathetic premotor neurons in the rostral ventrolateral medulla (RVLM) of the Sprague Dawley rat.

This population, approximately half of which are adrenergic C1 neurons (Stornetta, 2009), is a major source of the glutamatergic drive that maintains sympathetic vasomotor tone and therefore determines arterial blood pressure (Guyenet, 2006). The factors that determine the ongoing activity of these neurons have for several decades remained an unresolved core issue in the field of autonomic neuroscience (Coote, 2007; Guyenet et al., 2013): electrophysiological recordings from anesthetized animals suggest that RVLM sympathetic premotor neurons are a point of convergence for inputs from visceral and somatic reflex pathways (Brown and Guyenet, 1984; McMullan et al., 2008), from the central respiratory pattern generator (McAllen, 1987; Miyawaki et al., 1995; Verberne et al., 1999; Moraes et al., 2013), hypothalamus (Yang and Coote, 1998; Allen, 2002; Horiuchi et al., 2004; Korim et al., 2014), and higher centers including the prefrontal cortex and amygdala (Gelsema et al., 1989; Verberne, 1996), and that ongoing synaptic drive supports their activity (Lipski et al., 1996). However, the relative contributions of inputs from these regions, and in particular the level of input derived from local medullary neurons, has remained elusive.

Our strategy was first to use a recombinant herpes vector with a retrograde transduction profile, HSV-hCMV-YTB, to drive the expression of TVA, rabies glycoprotein, and a fluorescent reporter (YFP) in neurons that project to the interomediolateral cell column of the spinal cord (IML), a major site of termination of sympathetic premotor neurons. We then focally microinjected SADΔG(EnvA)-mCherry into the RVLM, selectively restricting its access to TVA-expressing bulbospinal neurons. This enabled us to map brain-wide sources of synaptic input to bulbospinal RVLM neurons using an image alignment tool based on the Waxholm volumetric atlas of the rat brain (Papp et al., 2014; Kjonigsen et al., 2015).

## Materials and methods

Experiments were approved by Macquarie University Animal Ethics Committee and conformed to the Australian Code of Practice for the Care and Use of Animals for Scientific Purposes.

### Vector preparation

#### SADΔG production

Rabies glycoprotein-transcomplemented SADΔG-mCherry (Wickersham et al., 2007a) and SADΔG(EnvA)-mCherry was produced and titrated as described by Osakada and Callaway (2013); the titers used for injections were 2 × 10^9^ and 6.8 × 10^7^ IU/ml respectively. SADΔG(EnvA)-mCherry purity was determined by infection of naïve HEK cells and determined to contain approximately 5.3 × 10^3^ unpseudotyped virions per ml. Injection of SADΔG(EnvA)-mCherry in the absence of YTB expression resulted in no labeling in two control experiments.

#### Retrograde HSV vectors

A recombinant herpes simplex type1 (HSV) vector with a retrograde tropism was used to drive expression of rabies glycoprotein, TVA, and YFP (HSV-hCMV-YTB). The gene cassette was derived from the pCAG-YTB plasmid (Addgene 26721) and cloned into recombinant HSV amplicons under the control of the human cytomegalovirus promoter. HSV-hCMV-YTB was supplied at 3 × 10^8^ IU/ml and diluted 1:4 with 0.9% saline containing blue fluorescent polystyrene spheres immediately prior to injection to mark the injection site (1:10,000, Thermo Scientific, Australia, 09980508). Control vectors that drive the expression of reporter proteins (HSV-hCMV-GFP, HSV-hCMV-mCherry) were used in initial experiments to determine the time course of protein translation and segment the anatomical boundary of the RVLM. Control vectors were used undiluted and animals sacrificed after 4–5 days.

### Vector injections

#### Spinal cord injections of retrograde herpes vectors

Adult male Sprague Dawley rats (165–500 g) were anesthetized with intraperitoneal ketamine (75 mg/kg; Parnell Laboratories, Australia) mixed with medetomidine (0.75 mg/kg; Pfizer Animal Health, Australia) and treated with prophylactic antibiotics (100 mg/kg Cephazolin sodium, i.m.; Mayne Pharma, Australia) and analgesia (2.5–10 mg/kg Carprofen, s.c.; Norbrook Pharmaceuticals, Australia). Two 500 nl injections of HSV-hCMV-YTB were made over 5–10 min at coordinates corresponding to the left T2 IML as previously described (Turner et al., 2013). Injections were separated by 1 mm rostrocaudally and the pipette was left in position after injections for approximately 5 min before its slow retraction. At the end of surgery anesthesia was reversed with atipamazole (1 mg/kg s.c., Pfizer Animal Health, Australia) and rats were observed until ambulatory and then returned to their home cages. Rats were monitored closely for the remainder of the experiment with additional analgesia as required. For experiments in which HSV-hCMV-GFP/mCherry control vectors were used the same general surgical approach was employed, but vector injections were made bilaterally at the T2 and/or T10 spinal cord.

#### Brainstem microinjections

One to five days after injection of HSV-hCMV-YTB rats were prepared for surgery as described above and positioned in a stereotaxic frame in the skull flat position. The left facial nucleus, an anatomical landmark directly rostral to the RVLM, was mapped using a micropipette containing SADΔG(EnvA)-mCherry by recording antidromic field potentials evoked by stimulation of the facial nerve (Turner et al., 2013). Fifty to seventy five nl of SADΔG(EnvA)-mCherry was microinjected 100–300 μm caudal to the facial nucleus at a depth equivalent to the ventral surface of the facial nucleus. The pipette was left in position for ~5 min prior to its withdrawal. Rats were treated as described above and allowed to recover for up to 7 days.

In initial experiments we observed histological signs of injury in HSV-hCMV-YTB-transduced neurons that were independent of rabies infection. Neurons developed a blebby appearance with retracted dendrites and spheroidal somata about 7 days after spinal injections, suggesting latent toxicity of the HSV vector. This effect was largely mitigated by dilution of HSV-hCMV-YTB.

We also found that longer intervals between HSV and rabies injections were associated with more off-target infection of bulbospinal (TVA-expressing) neurons outside of the RVLM (especially the paraventricular nucleus, midline raphe, rostral ventromedial medulla and C3 regions). Such neurons were easily identified by their dual expression of mCherry and YFP: contaminated experiments were excluded from analysis. Optimal results were obtained when SADΔG(EnvA)-mCherry was injected 24 h after HSV-hCMV-YTB and animals kept for a further 6 days.

### Histology

Animals were euthanized with sodium pentobarbital (>150 mg/kg, Lethabarb, Virbac, Australia) and immediately transcardially perfused with 300 ml ice cold heparinized saline followed by 300 ml 4% paraformaldehyde. The brain and thoracic spinal cord were then removed and post-fixed overnight. With the assistance of a brain matrix, brains were cut coronally 2 mm caudal to the olfactory bulb and mounted frontal pole down on a vibratome plate so that the ventral surface of the brain was approximately perpendicular to the plate. The entire brain was sectioned at 50 μm in the coronal plane using a Leica VT1200S vibrating microtome and collected in 4 bins in 0.01 M Tris-phosphate buffered saline (TPBS). Pot 1 was mounted directly onto glass slides to maintain section order. Brainstem sections from a second pot were processed for YFP and TH immunoreactivity so that starter neurons could be identified. The other pots were transferred to cryoprotectant solution (500 μM polyvinylpyrrolidone, 76.7 mM Na_2_HPO_4_, 26.6 mM NaH_2_PO_4_, 876 mM sucrose, 5 mM ethylene glycol) for storage at − 20°C.

#### Immunohistochemistry and *in situ* hybridization

Sections were permeabilized in TPBS containing 0.2% Triton-100 for 3 × 15 min and blocked for nonspecific binding in TPBS containing 2% bovine serum albumin and 0.2% Triton-100 for 1 h at room temperature. Primary antibodies (see Table 1) were added to the blocking buffer and sections were incubated for 48 h at 4°C. Sections were washed in TPBS 3 × 30 min and incubated in secondary antibodies for 12 h at 4°C. Processed sections were washed again in TPBS 3 × 30 min before being mounted on glass slides with Dako fluorescence mounting medium and cover slipped. ISH was conducted to examine double labeling of rabies-infected input neurons with GAD67 mRNA, a marker of GABAergic neurons, and GLYT2 mRNA, a marker of glycinergic neurons. ISH probes and processing were identical to those described by Bowman et al. (2013) and Le et al. (2016) respectively.

**Table 1:** **Antibody Table**.

**Type**	**Antigen**	**Species**	**Tag**	**Conc**.	**Clonality**	**Manufacturer**	**Cat No./RRID**
1°	TH	Mouse	N/A	1:1000	Mono	Sigma	T1299/AB_477560
1°	NK1R	Rabbit	N/A	1:1000	Poly	Sigma	S8305/AB_261562
1°	Vasopressin	Rabbit	N/A	1:1000	Poly	Millipore	AB1565/AB_11212336
1°	Choline Acetyltransferase (ChAT)	Goat	n/a	1:800	Poly	Chemicon, Millipore	AB144P/AB_2079751
1°	GFP	Rabbit	N/A	1:500	Poly	Life Technologies	A6455/AB_2313717
2°	Rabbit IgG (H+L chain)	Donkey	Alexa Fluor® 488	1:500	Poly	Life Technologies	A21206/AB_141708
2°	Rabbit IgG (H+L chain)	Donkey	Alexa Fluor® 647	1:500	Poly	Life Technologies	A31573/AB_10561706
2°	Mouse IgG (H+L chain)	Donkey	Alexa Fluor® 647	1:500	Poly	Life Technologies	A31571/AB_162542
2°	Sheep IgG (H+L chain)	Donkey	Alexa Fluor® 647	1:500	Poly	Jackson Immunoresearch	713-605-003/AB_2340750

### Imaging

For whole-brain mapping of rabies-infected neurons every 4th histological section lying between the cervical spinal cord and the most rostral section containing labeled neurons was imaged under epifluorescence (Zeiss AxioImager Z2 microscope, 10x/0.30 NA M27 objective lens running ZEN 2011). Images were obtained from sections that were immediately mounted during cutting, preserving order, except for sections from the RVLM region; these data were obtained from alternative sections that were processed for YFP and TH immunoreactivity. Neurons that contained both YFP and mCherry were classified as starter neurons and were further sub-classified as TH-immunoreactive (C1) or TH-negative (non-C1). Input neurons and C1/non-C1 starter neurons were manually annotated on each image using Zen software; the pixel coordinates of annotated neurons were then extracted from file metadata using the ImageJ/FIJI package (NIH, Bethesda, Maryland, USA) and tabulated.

Epifluorescence imaging at 10x permitted efficient imaging of whole-brain datasets at the expense of sensitivity, biasing against lightly labeled or small neurons. This was particularly problematic for detection of lightly-labeled TH-immunoreactive starter neurons; confocal reimaging (20x objective, Leica TCS SP5X) of three sections containing starter neurons previously imaged and analyzed as described above indicated that approximately a third of the C1 starter neurons detected under confocal had been identified as non-C1 under epifluorescence (19/27 vs. 13/27). This problem did not apply to detection of rabies-infected neurons, which were unambiguously labeled.

#### Image alignment and anchoring

**Figure 1:**
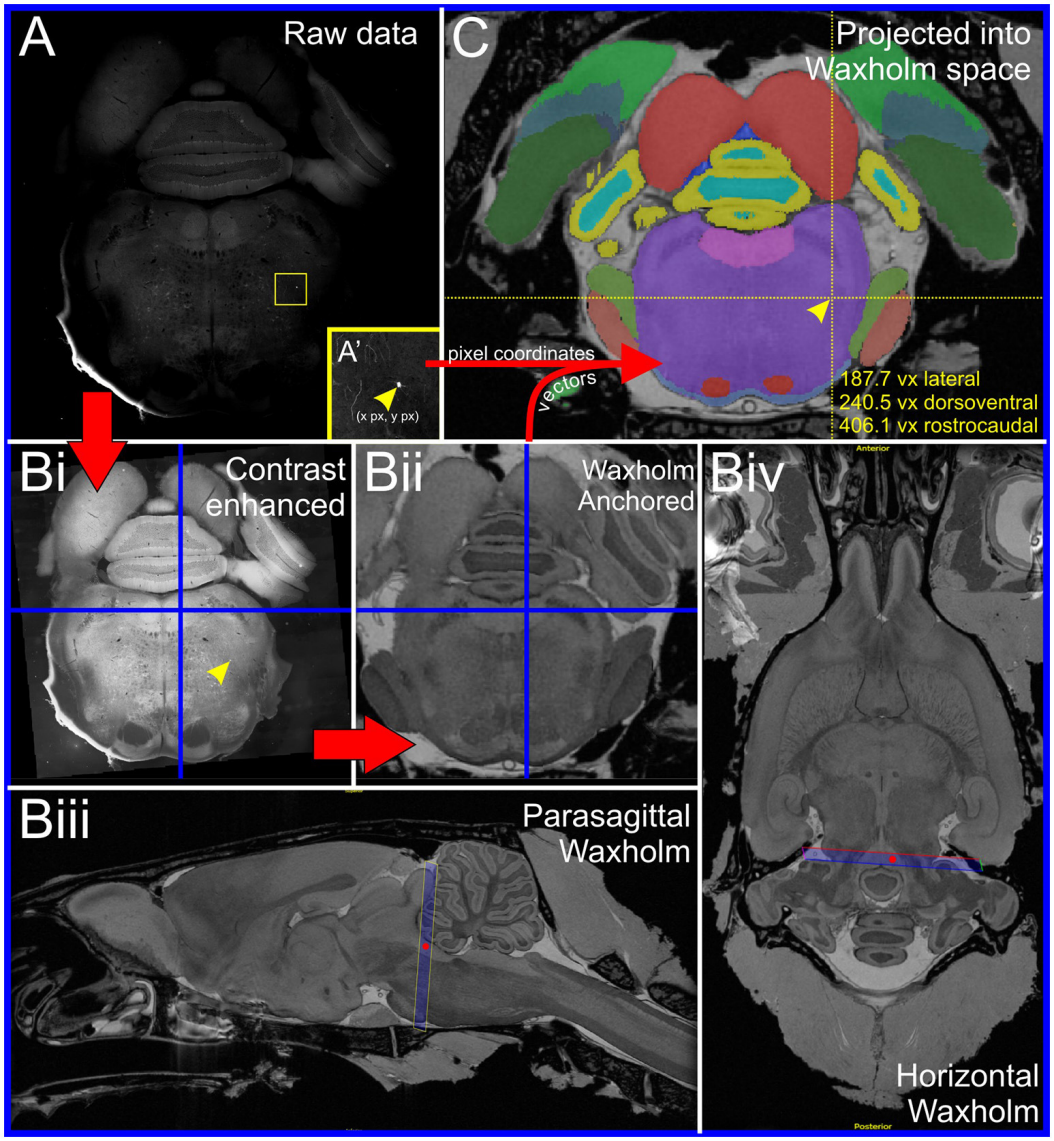
**Anchoring workflow. (A)** Original coronal epifluorescence image showing the location of a single rabies-labeled input neuron (yellow box, inset in **A'**). Following manual annotation, the pixel coordinates of the neuron were exported to a spreadsheet and the image contrast adjusted for optimal visualization of anatomical landmarks **(Bi)**. The image was then aligned to a corresponding section plane through the Waxholm atlas template **(Bii)** using a beta version of an image alignment/anchoring tool embedded in the Navigator-3 system. The anchoring tool allowed accurate positioning of the image plane in the MRI-derived atlas template. The orientation of the plane of the image is close to coronal, as shown in the blue frames in **(Biii)** and **(Biv)**. The parasagittal plane through the atlas template shown in **(Biii)** corresponds to the vertical blue line in **(Bi)** and **(Bii)**, whereas the horizontal plane in **(Biv)** corresponds to the horizontal blue lines in **(Bi)** and **(Bii)**. The red dots in **(Biii)** and **(Biv)** represent the intersections with the horizontal and parasagittal planes, respectively. Anchoring vectors generated by Navigator-3 were then used to translate the pixel co-ordinates of annotated neurons into xyz Waxholm coordinates and integrated into the Waxholm segmentation model **(C)**.

Image alignment and anchoring was achieved using a beta version of the AligNII tool embedded in Navigator-3 (N3), a web-based data management system currently under development of the University of Oslo Neuroinformatics group (see Figure 5 in Papp et al., 2016). An overview of the image alignment workflow is provided in Figure 1: microscope images were contrast-optimized for differentiation of gray and white matter and uploaded to N3 where the section images were overlaid onto virtual sections of the Waxholm atlas template, a whole brain MRI dataset obtained from a male Sprague Dawley rat (Papp et al., 2014). The cut angle of the MRI dataset was manually adjusted to match the histological section, allowing the user to align histological sections cut at any plane to the reference dataset and therefore compensating for deviations from standard cutting planes or tissue distortion. Once each image was optimally aligned to its MRI equivalent the image was considered “anchored”; geometric vectors corresponding to the rostrocaudal level of the image origin, deviation from the vertical and horizontal planes, scaling and rotation were calculated by the anchoring tool in the N3 platform and exported as metadata. With this information the position of any point in a histological image could be converted to 3-dimensional Waxholm coordinates in Microsoft Excel. Waxholm coordinates are by convention presented in the xyz format (lateral, rostrocaudal, dorsoventral) with a voxel resolution of 39 μm. The interested reader is directed toward CutNii, a freely downloadable explorer and custom-angle slicer for the Waxholm dataset (Csucs and Bjaalie, 2015), which is similar to the N3 tool used for image alignment (although it does not allow overlay or anchoring of histological images).

#### Volumetric brain modeling

The raw MRI data and corresponding segmentation model of the Waxholm Sprague Dawley rat were downloaded from the International Neuroinformatics Coordinating Facility Software Center (Papp et al., 2015) and imported into Imaris volumetric imaging software (Version 8.1, Bitplane AG, Switzerland) following conversion to the Biorad format in ImageJ. Each segmented area was individually rendered using the Imaris “contour surface” function, resulting in a surface-rendered model that incorporates regions demarcated in Waxholm space. Tabulated Waxholm coordinates of Input, non-C1 starter, and C1 starter neurons were then imported using a Python script (“*CreateSpotsFromFile*,” http://open.bitplane.com/tabid/235/Default.aspx?id=70), resulting in a 3d model of the Waxholm brain populated with points corresponding to identified neurons. Another script was then used to automatically quantify the number of input neurons that lay within each segmented region (“*Spots split into surface objects,”* http://open.bitplane.com/tabid/235/Default.aspx?id=19). The Imaris-rendered Waxholm brain (containing the RVLM connectomic dataset) is available as a supplementary download and can be used freely by other researchers (http://datadryad.org/resource/doi:10.5061/dryad.q5t5s). The dataset can also be viewed with the free Imaris SceneViewer program (www.bitplane.com). The voxel coordinates of starter and input neurons are included in tabulated format in Data Sheet 1.

#### Segmentation of the facial nucleus, RVLM, Bötzinger and pre-Bötzinger complex in Waxholm space

The Waxholm segmentation model does not differentiate brainstem subnuclei, so we used the locations of bulbospinal TH-immunoreactive RVLM neurons as the basis for segmentation of the RVLM. Data were obtained from six rats in which neurons projecting to the T2 and/or T10 spinal segments were retrogradely labeled by HSV-hCMV-GFP or -mCherry control vectors. Vector injections and histology were conducted as described above and 273 TH-positive bulbospinal RVLM neurons were annotated and anchored in Waxholm space. The lateral coordinates of all neurons were represented as being on *both* sides of the brainstem for segmentation; two-dimensional contour maps indicating the density of labeling were then generated for each dorsoventral level using the Plotly visualization tool (https://plot.ly, 10 voxel resolution). Contours enclosing pixels that contained >2 neurons/10 pixel radius in the horizontal plane were converted to an image stack, imported into the virtual Waxholm rat brain using Imaris, and surface rendered to define the boundaries of the RVLM (Video 1).

The extent of the facial nucleus was annotated directly from the Waxholm MRI dataset in Imaris; the Bötzinger and pre-Bötzinger Complex were defined as longitudinal cylinders, 500 μm in diameter that ran center-aligned and immediately ventral to nucleus ambiguus. The Bötzinger region was defined as starting at the caudal pole of the facial nucleus and running 600 μm caudal; the pre-Bötzinger Complex was defined as running between 700 and 1200 μm caudal to the facial nucleus (Le et al., 2016).

### Cluster analysis

*K*-means analysis was used to partition groups of input neurons based on their 3d distribution. The algorithm determines clusters by assigning data points to a closest mean (centroid), assigned initially at random and iteratively refined as data points are sequentially accumulated within a cluster. Analysis was performed and density plots generated in “R project” (R Foundation for Statistical Computing, Vienna, Austria, 2005, http://www.r-project.org). The voxel coordinates of input neurons segregated by cluster are provided in Data Sheet 2.

## Results

### Retrograde transduction of spinally projecting neurons and segmentation of the RVLM in Waxholm-space

Microinjection of HSV vectors at the thoracic spinal cord drove reporter expression in bulbospinal neurons within 12 h, with maximal expression apparent by 2–4 days. Reporter-labeled neurons spanned the RVLM, rostral ventromedial medulla (RVMM), and midline raphe region, and were identified in other sympathetic premotor nuclei such as the C3, paraventricular, A5, and locus coeruleus (Supplementary Image 1).

The distribution of TH-immunoreactive neurons labeled by HSV control vectors was plotted in Waxholm co-ordinates (Figure 1) and was used to define the anatomical boundaries of the RVLM (Supplementary Image 1). The geometric epicenter was located at Waxholm coordinates 198 lateral, 313 rostrocaudal, 182 dorsoventral (Supplementary Images 1D–F), corresponding to a position 1.78 mm lateral to the midline, 117 μm rostral to the caudal pole of the facial nucleus, and 339 μm dorsal to the ventral surface of the medulla immediately beneath the epicenter. The segmentation defined a dorsoventrally flattened ovoid (Supplementary Images 1G–I) in which the long axis runs medial in more rostral sections and spans Waxholm coordinates 176–218 lateral (2.6–1 mm lateral to midline), 287–327 rostrocaudal (900 μm caudal to 663 μm rostral to caudal pole of the facial nucleus) and 174–198 dorsoventral (within 897 μm from the ventral surface of the brainstem). This region contained 86% of TH-positive bulbospinal neurons used to generate the segmentation (*n* = 236) and corresponds well with the pressor region that encompasses the rostral RVLM and perifacial zone (Goodchild and Moon, 2009).

### Monosynaptic tracing was predominantly restricted to putative RVLM sympathetic premotor neurons

**Figure 2:**
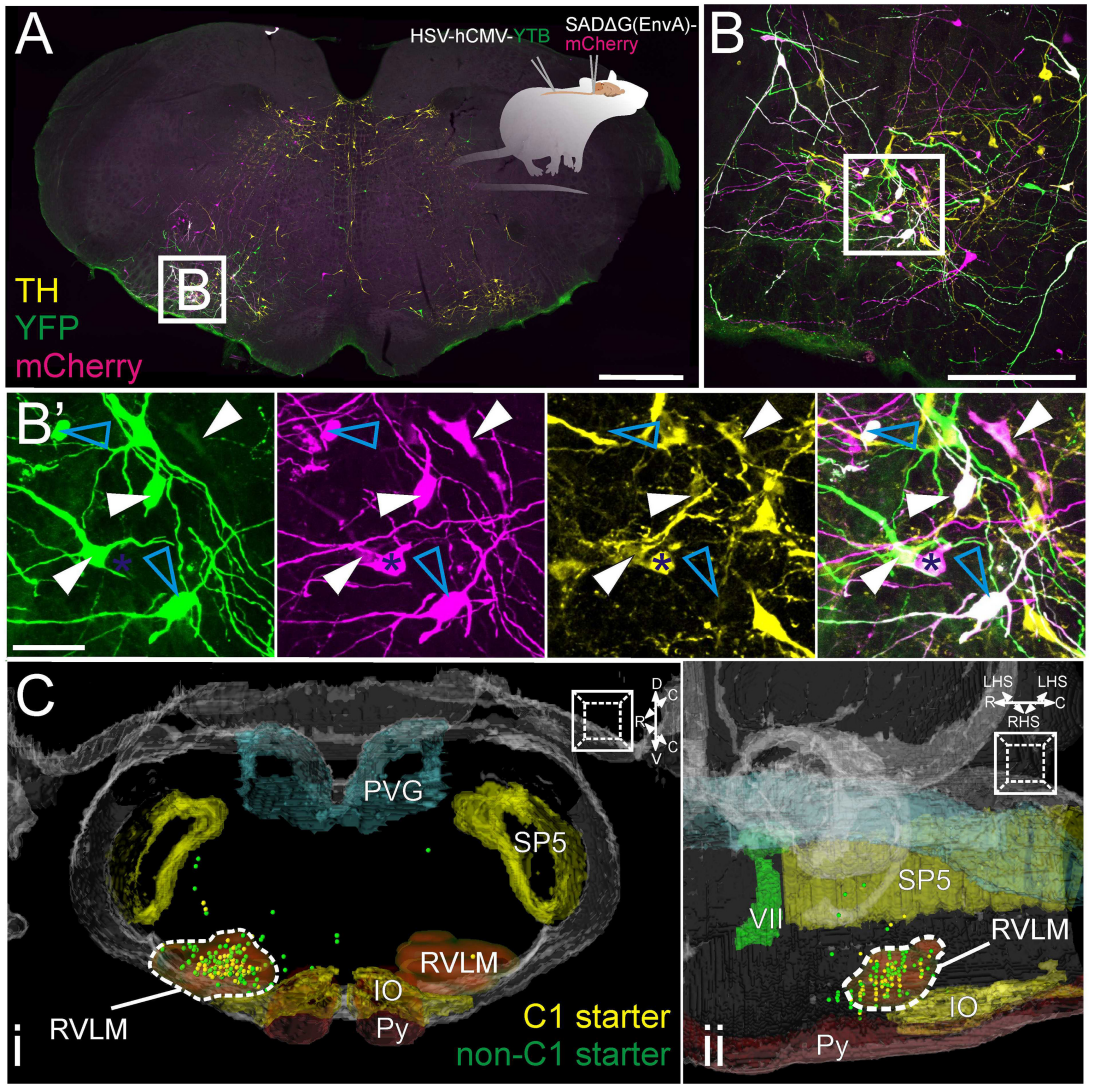
**Experimental strategy (inset in A):** HSV-hCMV-YTB was injected at the T2 IML, retrogradely driving the expression of TVA, rabies glycoprotein, and YFP in spinally projecting neurons. SADΔG(EnvA)-mCherry was subsequently injected into the RVLM, exclusively infecting neurons that express TVA, and seeding trans-synaptic infection of monosynaptically connected input neurons. **(A)** Coronal brainstem section at the level of the rostral ventrolateral medulla (RVLM) processed for immunoreactivity to TH and YFP; starter neurons are defined by the expression of both YFP and mCherry. **(B)** Confocal detail with magnified **(B')** examples of C1 (solid arrowheads) and non-C1 (cyan arrowheads) starter neurons (individual channels and merged image). Blue asterisk indicates a C1 input neuron. **(C)** Distribution of C1 (yellow) and non-C1 (green) starter neurons from four experiments plotted in Waxholm space, shown from coronal **(Ci)** and parasagittal **(Cii)** perspectives. Anatomical landmarks are Waxholm-segmented boundaries of the inferior olive (IO), spinal trigeminal nucleus (SP5), RVLM, pyramidal tract (Py), facial nerve (VII), and periventricular gray (PVG). Scale bars 1000 μm **(A)**, 250 μm **(B)**, and 100 μm **(B')**.

Data from 4 animals were selected for detailed connectomic analysis. As illustrated in Supplementary Image 2, unilateral injection of HSV-hCMV-YTB directed at the second thoracic (T2) IML resulted in retrograde transduction of bulbospinal neurons in the ventrolateral medulla, the majority of which were ipsilateral to the injection site (82% (71–88) [mean (range), *n* = 4 rats]). Subsequent injection of SADΔG(EnvA)-mCherry in the RVLM resulted in the primary infection of bulbospinal RVLM neurons. As shown in Figure 2, bulbospinal “starter” neurons were identified by their co-expression of YFP and mCherry and many were immunoreactive for TH (Figure 2B').

Neurons were counted on every fourth section: on average 38 starter neurons were identified per animal (range: 19–67), of which 40% (30–45) were confirmed as TH-positive C1 neurons under epifluorescence (which underrepresents the proportion of C1 starter neurons by up to 50%: see Methods–Imaging). Seventy eight percent (74–87%) of starter neurons lay within the Waxholm RVLM boundary defined above (Figure 2C). Ectopic starter neurons that fell outside the RVLM were observed around the lateral and dorsal perimeter of the facial nucleus and adjacent sympathetic premotor nuclei which included the rostral ventromedial medulla (RVMM), caudal raphe and contralateral RVLM. Video 1 shows the Waxholm MRI dataset overlaid with the distribution of C1 and non-C1 starter neurons from 4 rats subjected to detailed analysis, along with the boundary of the RVLM region.

### Inputs to putative RVLM sympathetic premotor neurons predominantly arise from medullary nuclei

**Figure 3:**
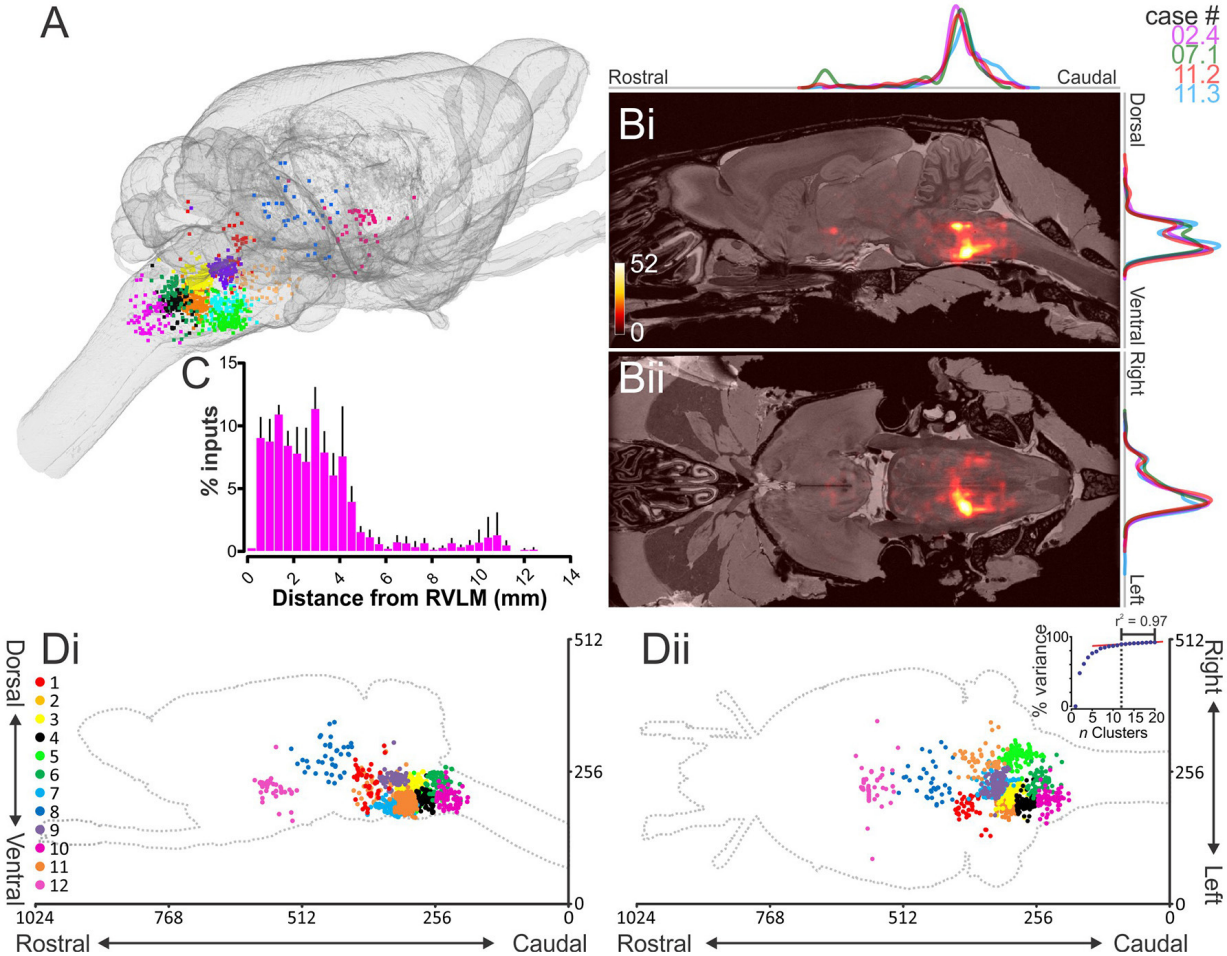
**Distribution of monosynaptic input neurons. (A)** Input neurons to putative sympathetic premotor neurons in the rostral ventrolateral medulla (RVLM) segregated into 12 distinct clusters and plotted in the Waxholm volumetric rat atlas. **(B)** Heat maps showing input neuron density overlaid on the Waxholm MRI dataset in the parasagittal **(i)** and horizontal **(ii)** planes. Scale indicates number of neurons per 10 voxels. Normalized plots of input densities for individual experiments are plotted alongside heat maps. **(C)** Absolute distance of input neurons from RVLM epicenter (*n* = 4 rats). **(D)** Cluster plots presented in the sagittal **(i)** and horizontal **(ii)** planes. Inset in **(Dii)** shows the proportion of total variance accounted for by incremental increases in cluster number; the turning point of the curve, after which slope becomes linear, occurs at 12 clusters.

Monosynaptic input neurons were identified throughout the brain as mCherry, non-YFP neurons. 1298 input neurons were identified in total [325 (220–561) per animal], corresponding to an input: starter ratio of 9.8 (4.4–13.4). The overall distribution of input neurons is shown in Figures 3A,B: although the absolute number of input neurons and efficiency of trans-synaptic spread was variable, the overall pattern of inputs was consistent between animals: input neurons were encountered from the most caudal point quantified, the cervical medullary junction, to the hypothalamus at the level of the optic chiasm, with a pronounced [77% (75–80)] ipsilateral bias. Nodal edge-length analysis (the shortest distance between each neuron and the epicenter of the RVLM) indicates that most input neurons lie in close proximity to the RVLM, with 50% of inputs residing within 2.5 mm (2.3–2.7) with a progressively diminishing proportion of inputs identified at increasing distances: only 10% of monosynaptic input neurons lie more than 5 mm from the RVLM (Figure 3C). Input neurons were also distributed throughout the thoracic and cervical spinal cord, although these data were not quantified.

**Figure 4:**
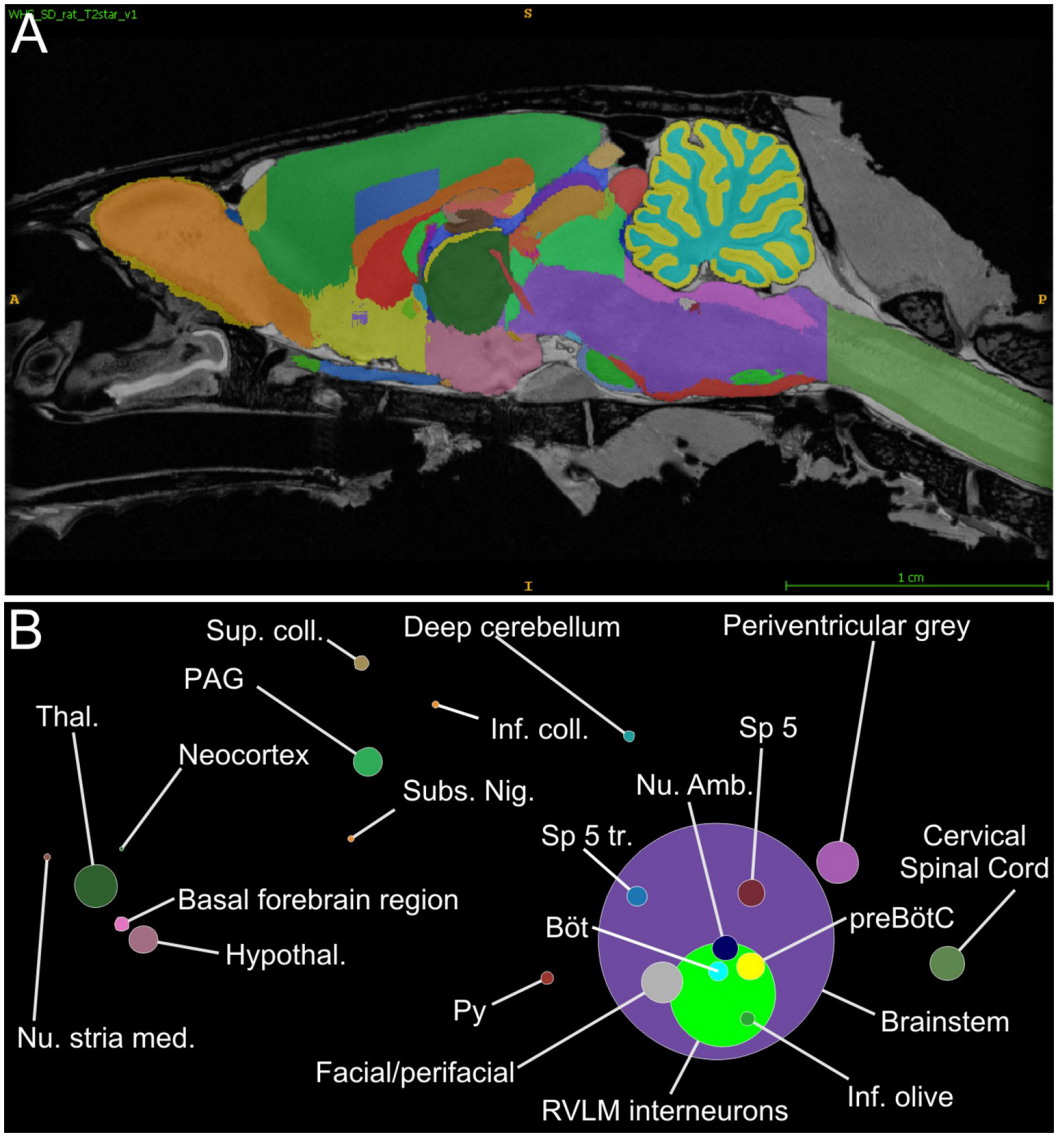
**RVLM input neurons organized by Waxholm segment. (A)** Parasagittal view through the Waxholm volumetric atlas for reference; different colors indicate different brain segments. **(B)** Proportions of input neurons per Waxholm segment (summed from 4 experiments) colored and positioned according to their corresponding Waxholm regions. The diameter of each circle corresponds to the number of inputs therein. See Table S2 for source data and abbreviations.

A script that automatically counts the number of neurons within each region of the Waxholm atlas was used to quantify the regional distribution of input neurons. These data are graphically represented in Figure 4 and tabulated in Table S1: the overwhelming majority of inputs resided within the Waxholm-defined boundary of the brainstem 92% (88–94), with inputs arising from the forebrain and midbrain accounting for 3% each and the remainder originating in the cervical spinal cord and cerebellum. Sub-nuclei of the brainstem are not well represented in the current iteration of the Waxholm atlas; of those regions thus far defined the highest source of input was the RVLM itself, which contained 14% of input neurons.

**Figure 5:**
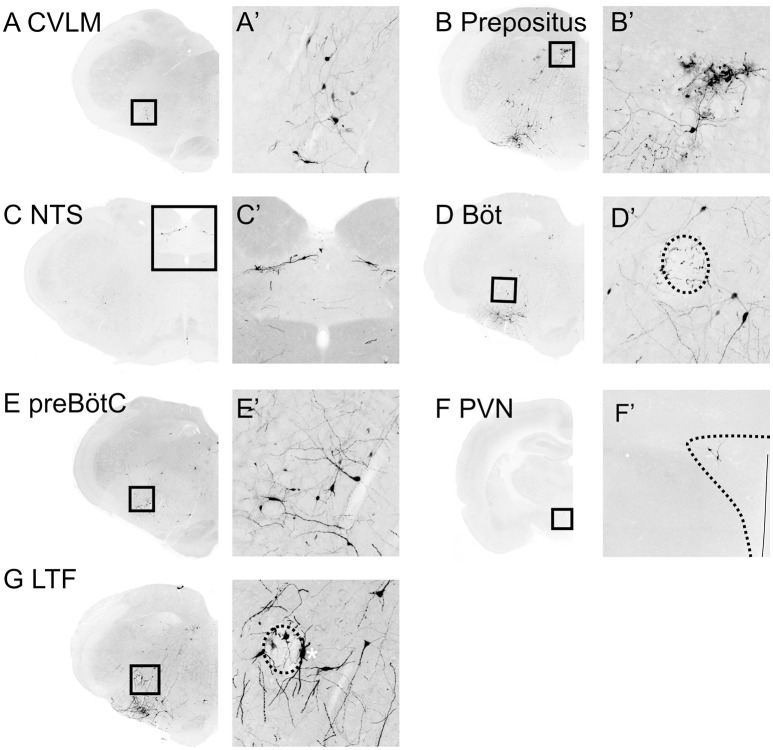
**Medullary inputs to RVLM sympathetic premotor neurons**. Inverted epifluorescence micrographs with high power insets **(A'–F')** of boxed regions illustrating monosynaptic inputs from the caudal ventrolateral medulla (CVLM: **A**), nucleus prepositus **(B)**, nucleus of the solitary tract (NTS: **C**), Bötzinger (Böt: **D**), pre-Bötzinger Complex (preBötC: **E**), paraventricular nucleus of the hypothalamus (PVN: **F**) and lateral tegmental field (LTF: **G**). Nucleus ambiguus is indicated by the hatched ovals in **(D,G)**. ^*^in Panel **(G)** indicates a TH-positive starter neuron.

**Figure 6:**
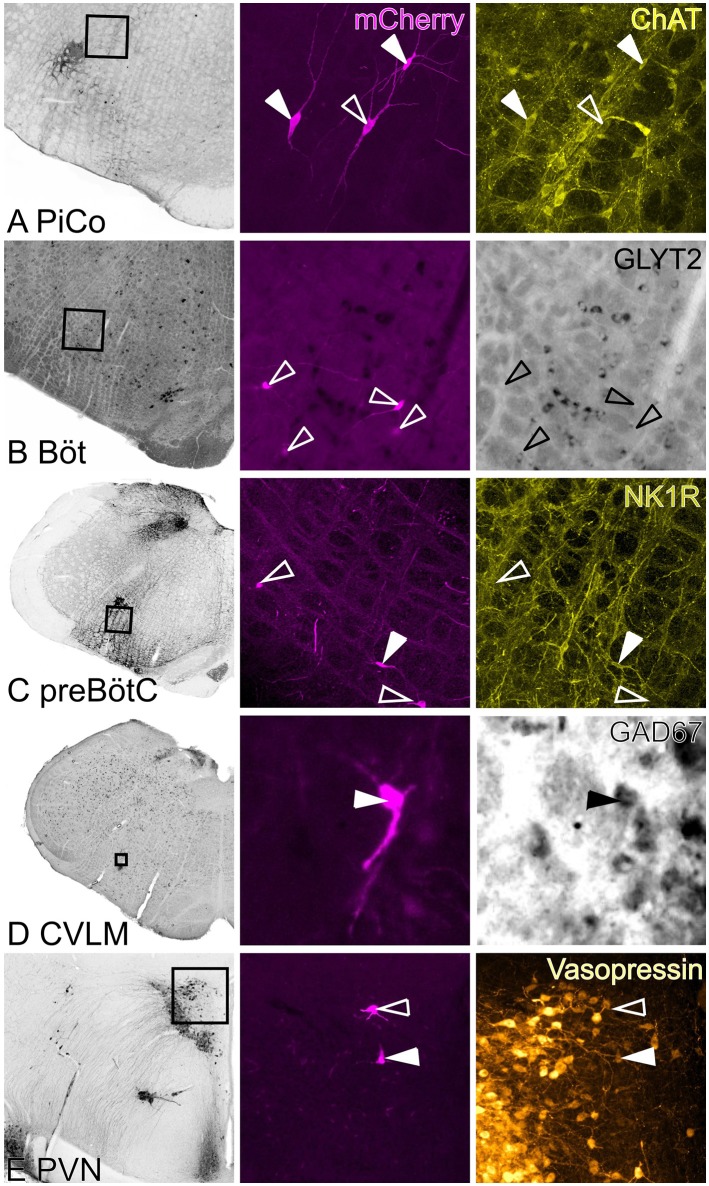
**Neurochemical phenotypes of input neurons**. Left hand panels are low power images of *in situ* hybridization/immunohistochemistry indicating region shown in high power images. Middle panel shows rabies-labeled input neurons, right panels show *in situ* hybridization/immunofluorescence. Closed arrowheads indicate double-labeled neurons; open arrowheads indicate the positions of rabies labeled input neurons. **(A)** ChAT-immunoreactive input neurons in the region of the lateral reticular formation that corresponds to the mouse PiCo. **(B)** Bötzinger input neurons were abundant but none were identified as GlyT2-positive. **(C)** NK1R-positive and -negative pre-Bötzinger Complex inputs. **(D)** GAD67-positive input neurons in the CVLM. **(E)** Vasopressin-positive and -negative PVN inputs. PiCo: post-inspiratory complex, Böt: Bötzinger, preBötC: pre-Bötzinger Complex, CVLM: caudal ventrolateral medulla, PVN: paraventricular nucleus.

*K*-means analysis was used to objectively group input neurons based on their spatial distribution. We empirically determined that 12 was an appropriate number of clusters (*k*) by which to partition the dataset by plotting the percentage of variance explained by clusters generated for *k*-values 1–20 and identifying a point at which the increase in explained variance was marginal for further increases in *k*, denoted by an “elbow” in the curve (Figure 3Dii, inset). The 12 clusters identified accounted for 89.3% of variance in the dataset (Figure 3); a detailed overview of identified clusters and their correspondence to the literature is presented in Table S2. Eight of 12 input clusters were located within the medulla, the largest of which (Cluster 2) encompassed the ipsilateral RVLM (including occasional trans-synaptically labeled C1 neurons: Figure 2B), Bötzinger region (Figure 5D), nucleus ambiguus (Figure 5G), and choline acetyltransferase (ChAT)-immunoreactive cells in a region of the ventral lateral tegmental field (Figure 6A) that may represent the rat analog of the post-inspiratory complex (PiCo: Anderson et al., 2016). In a sample of 21 input neurons identified as lying within the Bötzinger region using previously published criteria (Le et al., 2016), none contained glycine transporter 2 mRNA, a marker for respiratory function in this region (Figure 6B, Schreihofer et al., 1999). Adjacent clusters enveloped the dorsal LTF (Cluster 3) and pre-Bötzinger Complex (Cluster 4, Figure 5E), including neurokinin-1 receptor (NK1R) immunoreactive neurons (Figure 6C), a putative marker for respiratory function in this region (Gray et al., 2001). Cluster 4 also spanned the rostral ventral respiratory group and caudal ventrolateral medulla (CVLM: Figure 5A), including confirmed GABAergic CVLM neurons (Figure 6D). Input clusters also included the rostral ventromedial medulla and caudal Raphe nuclei (Cluster 7) and a distinct input from the region immediately ventral to the nucleus prepositus hypoglossi (Cluster 9, Figure 5B). Other brainstem clusters spanned the intermediate and commissural nucleus of the solitary tract (NTS: Figure 5C, Cluster 6), the caudal pressor area and caudal ventral respiratory group (Cluster 10), and the ventral aspect of the contralateral medulla (Cluster 5). A flythrough of the entire dataset, projected onto the MRI Waxholm dataset and segregated by color into its component clusters, is presented in Video 2.

### Supramedullary nuclei constitute a minor source of input to RVLM sympathetic premotor neurons

Supramedullary inputs included diffuse pontine clusters that encompassed the ipsilateral (Cluster 1) and contralateral (Cluster 11) A5 adrenergic cell group, subcoeruleus, Kölliker-Fuse, and medial and lateral parabrachial nuclei. The single midbrain cluster (Cluster 8) incorporated the lateral and ventrolateral periaqueductal gray (PAG) and colliculi, and a hypothalamic cluster (Cluster 12) composed from neurons residing within the paraventricular hypothalamic nucleus, including confirmed vasopressinergic neurons (Figures 5F, 6E) and neurons in the lateral and perifornical hypothalamic areas.

## Discussion

The objective of the current study was to definitively and quantitatively map the afferent connectome of putative RVLM sympathetic premotor neurons, a regulatory axis through which convergent sensory and limbic inputs are thought to summate to produce baseline sympathetic nerve activity. We identified prominent inputs from well-established neurohumoral and viscero-sympathetic actuators such as the area postrema and NTS, and inputs that spanned medullary autonomic (A5, RVLM, ventromedial medulla, CVLM, LTF) and respiratory nuclei (Bötzinger, pre-Bötzinger Complex, PiCo, rostral ventral respiratory group), as well as supramedullary autonomic nuclei (paraventricular nucleus, ventrolateral PAG, lateral hypothalamus) (Dampney, 1994b; Guyenet, 2006; Card et al., 2011; Stornetta et al., 2016). However, the most striking feature of the connectome is its diffuse distribution, which contrasts with the nodal ball-and-stick schemes sometimes used to conceptualize it (Dampney, 1994a; Pilowsky and Goodchild, 2002; Guyenet, 2006), and its strong weighting toward local inputs, with around 14% of input neurons residing within the RVLM region and 30% of inputs lying within 1 mm of the RVLM epicenter. In contrast, the dataset includes a relatively low number of inputs from the forebrain (3%) and midbrain (3%), and a virtual absence of inputs from locus coeruleus, the amygdala, cortex and subfornical organ and median preoptic nucleus, despite compelling prior evidence for both functional and neuroanatomical connectivity (Dampney et al., 1987; Cassell and Gray, 1989; Gelsema et al., 1989; Verberne, 1996; Saha, 2005; Card et al., 2011; Bou Farah et al., 2016). This pattern of connectivity is consistent with qualitative data from in a similar study that targeted TH-synthesizing RVLM neurons in the mouse (Stornetta et al., 2016) and supports the argument that RVLM sympathetic premotor neurons play little, if any, role in the generation of sympathoexcitatory responses to acute psychological stress or conditioned fear (Dayas et al., 2001; Carrive and Gorissen, 2008; Furlong et al., 2014; Dampney, 2015), although it does not rule out the possibility that input from cortical and limbic structures could be indirect, perhaps involving disynaptic relays through the abundant monosynaptically connected RVLM interneurons identified in this study. Alternatively, as the current study focused exclusively on inputs received by sympathetic premotor neurons that project to the T2 thoracic spinal cord, it could be that higher centers provide a more (numerically) significant input to RVLM neurons that project to other spinal segments.

The minor input received from supramedullary centers is unlikely to represent a limitation of SADΔG(EnvA), as distance is not thought to impair its labeling efficiency (Callaway and Luo, 2015; Schwarz et al., 2015). Furthermore, inputs from the amygdala and cortex were observed in control experiments using rabies glycoprotein-transcomplemented SADΔG-mCherry and retrograde herpes vectors, and have been reported in studies that used classical chemical tracers (Bowman et al., 2013; Bou Farah et al., 2016), confirming that these structures innervate the RVLM region; apparently just not those neurons targeted in the current study. Taken at face value, these data suggest that inputs from numerous local brainstem structures predominate in their capacity to influence sympathetic nerve activity compared to more distant regions, with the caveat that identification of monosynaptically connected neurons does not necessarily denote activity of those inputs: rabies spread is a function of synaptic strength, not synaptic activity (Ugolini, 1995; Brennand et al., 2011).

RVLM sympathetic premotor neurons include both C1 and non-C1 neurons, which differ in their functional and neurochemical phenotypes (Schreihofer and Guyenet, 1997; Stornetta et al., 2001) and may play differing roles in the generation of baseline sympathetic nerve activity and the elaboration of sympathetic reflexes (Schreihofer and Guyenet, 2000; Schreihofer et al., 2000; Madden et al., 2006; Burke et al., 2011). We used a herpes vector with a retrograde tropism to transduce putative sympathetic premotor neurons based on their axonal trajectory, allowing us to target both C1 and non-C1 bulbospinal neurons. One potential limitation of this approach is that SADΔG(EnvA)-mCherry injected into the RVLM could have theoretically infected TVA-expressing spinally-projecting neurons from other brain regions, either as a result of trans-synaptic spread (to spinally projecting neurons that provide collateral input to RVLM sympathetic premotor neurons) or by direct infection of axons that traverse the RVLM. Evidence of such “ectopic” starter neurons was apparent in pilot experiments, but was largely eliminated by limiting the interval between herpes and rabies injections to 24 h and by diluting the herpes vector. We speculate that TVA is differentially expressed on the somata but not the axons of herpes-transduced neurons at 24 h, accounting for the selective accessibility of RVLM neurons at this time-point, and conclude that bulbospinal RVLM neurons do not receive collateral input from other spinally projecting neurons because we did not see any evidence of ectopically infected spinally projecting neurons in other brain regions.

A notable deviation from the standard autonomic circuit model is the discovery of a conspicuous input from the region of the prepositus hypoglossi, a brainstem nucleus conventionally associated with vestibulo-occulomotor integration (McCrea, 1988) but also previously shown to drive pressor responses to glutamate microinjection (Talman and Robertson, 1991). Rabies-labeled prepositus neurons reside along the axonal trajectory of RVLM bulbospinal neurons (Lipski et al., 1995; Stornetta et al., 2016) but are not labeled by traditional retrograde tracers injected at the RVLM pressor region (Dampney, 1994b) (or retrograde rabies/herpes vectors—data not shown): we speculate that trans-synaptic infection of this population may have been via axoaxonic contacts or via the distal dendrites of starter neurons. Future functional studies will be required to elucidate the functional significance of this input.

Our dataset provides unequivocal evidence of monosynaptic inputs from respiratory subnuclei, providing clues to the neuroanatomical substrate responsible for respiratory-sympathetic coupling, the entrainment of sympathetic nerve activity to the phasic bursting of the central respiratory rhythm generator. Although direct interaction between respiratory and cardiovascular neurons in the brainstem has long been suspected (reviewed by Pilowsky et al., 1996; Taylor et al., 1999; Zoccal et al., 2014), technical difficulties have until now precluded unambiguous examination of this hypothesis. We found no evidence that input neurons in the Bötzinger region were glycinergic, (a functional marker of Bötzinger respiratory neurons: Schreihofer et al., 1999; Ezure et al., 2003), undermining the hypothesis that Bötzinger neurons are a source of inhibitory input to sympathetic premotor neurons (Sun et al., 1997). This is consistent with the observation that blockade of RVLM glycinergic transmission does not alter respiratory-sympathetic coupling (Guyenet et al., 1990), and suggests that the close appositions identified by Sun et al. (1997) may not represent functional synapses, an acknowledged limitation of light microscopy for reliable identification of synaptic contacts (Murphy et al., 1995; Descarries and Mechawar, 2000).

On the other hand, we identified pre-Bötzinger Complex input neurons that were immunoreactive for NK1R, a putative marker of glutamatergic inspiratory neurons in this region (Gray et al., 2001; Guyenet and Wang, 2001; Stornetta et al., 2003), providing a plausible structural basis for the inspiratory-locked sympathoexcitation evident in some sympathetic outflows (reviewed by Häbler et al., 1994; Pilowsky et al., 1996) and RVLM sympathetic premotor neurons (McAllen, 1987; Moraes et al., 2013). We also identified inputs from ChAT-immunoreactive neurons in the region of the LTF that may correspond to the PiCo, a group of glutamatergic cholinergic neurons recently reported to underlie the generation of postinspiratory activity in the mouse (Anderson et al., 2016). Although functional studies of the PiCo are yet to be replicated in the rat, we tentatively hypothesize that excitatory input from its analog could underlie the prominent post-inspiratory activity seen in rat lumbar and splanchnic sympathetic nerve activities (Haselton and Guyenet, 1989; Burke et al., 2010; Korim et al., 2012).

Few input neurons were found in the medullary retrotrapezoid/parafacial region that lies close to the ventral brainstem surface immediately adjacent to the RVLM. As this population is not thought to directly contribute to respiratory-sympathetic coupling (Moraes et al., 2012) their neurochemical phenotype was not studied in detail: such inputs that were identified did not conform to the small fusiform morphology or reticular distribution typical of RTN CO_2_-sensing neurons (Stornetta et al., 2006).

Like other mesoscale connectomes, the RVLM connectome is diffuse and highly non-uniform in its distribution. This level of complexity makes it difficult to conceptualize as a whole, and makes identification of its major subdivisions susceptible to operator bias. Principle component analysis (cluster analysis) provides a simple and unbiased way to do this but suffers some potential drawbacks. First, selection of the appropriate number of clusters (the value of *k*) is inexact; it can be estimated by examining the degree of variance accounted for by iterative increases in *k* and identifying the “elbow” after which the relationship between *k* and variance becomes linear (Thorndike, 1953: in the current study *k* = 12). However, as this value is a function of the variance of the dataset, one would expect *k* to increase if more experimental data were added—in other words, if one analyzed 10,000 input neurons instead of 1,000, the sensitivity of the analysis would be higher and it would resolve clusters not currently detectable. Second, cluster analysis was performed on the pooled connectomic dataset, rather than on each individual experiment. Although the overall normalized distribution of inputs was reproducible between experiments, this means that experiments in which a large number of input neurons were detected influence cluster detection more than those in which fewer inputs were detected. Taken together, cluster analysis of connectomic datasets should be seen as a useful platform for breaking complicated datasets down into a readily digestible overview, rather than a definitive structural analysis tool.

Overall, the spatial distribution of the RVLM connectome adheres to two key organizational principles evident when examining interregional connectivity profiles [i.e., at the macroscopic resolution: reviewed by Bullmore and Sporns (2012)]. The first is reciprocity, that is, regions innervated by the collateral branches of putative sympathetic premotor neurons such as the NTS, RVLM, CVLM/A1, raphe and PAG (Card et al., 2006; McMullan and Pilowsky, 2012; Stornetta et al., 2016) all in turn provide afferent input to RVLM sympathetic premotor neurons. The second is spatial embedding: RVLM sympathetic premotor neurons are more likely to receive input from nearby neurons than distant ones. The spatial embedding evident in the rat RVLM connectome is in striking accordance with the nodal edge-length distribution of the mouse mesoscale connectome, the most detailed whole brain connectivity atlas currently available (Oh et al., 2014), and in particular the spatial distribution of reciprocally connected nodes (Henriksen et al., 2016) (see Supplementary Image 3 for direct comparison). This supports the proposition, reviewed by Bullmore and Sporns (2012), that highly conserved general principles likely govern circuit topography irrespective of species, brain region, cell type or scale, and provides for the first time cell-type specific connectomic data that can be used to test general brain connectivity models.

Cell-specific connectome tracing provides unique insights into the organization of circuits that control populations of neurons selected based on their genetic, functional, or projection profiles (Yonehara et al., 2013; Pollak Dorocic et al., 2014; Pollock et al., 2014; Wertz et al., 2015). However, one of the challenges to this approach lies in the analysis and presentation of the data. A common strategy is to align histological images with corresponding plates from a stereotaxic atlas, count the number of neurons that reside within each segmented region, and then tabulate the output (Pollak Dorocic et al., 2014; Schwarz et al., 2015). Although superficially straightforward, we found that the reliability of this approach depends greatly on the expertise of the operator and can be compromised by even small misalignments in cutting plane. We calculate that each degree in deviation in the lateral plane results in an offset of ~280 μm rostrocaudal at the widest point of the rat brain, (which is further exaggerated by any deviation in the dorsoventral plane). Perfect histological cutting planes are virtually impossible to achieve, and so data presented in this way are intrinsically error-prone. The longevity of data presented as #cells/region are further undermined by the evolving taxonomy of the brain, variation in segmentation and nomenclature used by different reference atlases, and the ongoing discovery of new cell groups. An alternative approach is to present imaging data in its entirety, either as an online repository of still (Pollak Dorocic et al., 2014) or video images (Stanek et al., 2014). This allows other investigators to access the raw data, but independent analysis is hampered by the enormous volume of imaging data and the time commitment required to reanalyze it.

Our approach sidesteps both of these limitations: alignment of a 3d reference atlas to the histological image (rather than the other way around) eliminates potential errors caused by imperfect cutting angle or tissue distortion. Second, the compact size (current study <100 kb) and standardization of the positional metadata make it amenable to sharing and independent reanalysis, and eliminates the need for other investigators to directly interact with cumbersome raw imaging data (current study >300 Gb). Routine publication of such metadata would enable researchers to present complex neuroanatomical datasets in a standardized and transparent format that improves utility and reproducibility.

The promise of the connectomic approach is reflected by its widespread adoption and the continued refinement of connectome-tracing viral vectors (Osakada et al., 2011; McGovern et al., 2015; Kim et al., 2016; Reardon et al., 2016; Zingg et al., 2017), but tools that compile and quantify connectomic datasets remain primitive. The approach described here allows the quantification, standardization, and sharing of complete neuroanatomical datasets, and therefore provides a platform by which researchers may independently visualize, analyze, and compare each other's data. Bioinformatics at a single-cell resolution may allow researchers to move beyond the ball-and-stick diagrams often used to conceptualize the organization of spatially diffuse real-world neuronal networks (Rockland, 2015) and realize the full potential of novel connectomic technologies.

## Author contributions

Conceptualization: SM, AG, and AA; Methodology: BD, RN, and SM; Software: JB; Formal analysis: SL, BD, AT, and SM; Investigation: BD, AT, SL, CM, and SM; Resources: RR, PB, RN, and JB; Data curation: SL, BD, and SM; Writing—Original Draft: BD, AG, and SM; Writing—Review and Editing: All authors; Visualization: BD and SM; Supervision: SM; Project Administration: SM; Funding Acquisition: SM, AA, and AG.

### Conflict of interest statement

The authors declare that the research was conducted in the absence of any commercial or financial relationships that could be construed as a potential conflict of interest.

## Supplementary material

Supplementary Image 1 **Segmentation of the RVLM in Waxholm space. (A)** Experimental strategy: HSV vectors encoding GFP or mCherry were microinjected into the T2 or T10 spinal cord and **(B)** retrogradely transduced RVLM neurons (boxed area) were classified as C1 or non-C1; **(C)** shows examples of C1 neurons labeled from the T10 segment (open cyan arrowhead) and from both the T2 and T10 segments (closed white arrowhead). **(D)** Waxholm coordinates of 273 bulbospinal C1 neurons were plotted as 2d heat maps at each horizontal plane (detail in **D'**): the epicenter is indicated by crosshairs in the corresponding horizontal **(D)**, coronal **(E)**, and parasagittal **(F)** sections through the Waxholm MRI dataset. **(G–I)** Coordinates of individual bulbospinal C1 neurons (green spheres) and segmentation that enclosed 86% of neurons (green surface) plotted in 3d model of the Waxholm brain. Regional landmarks are the facial nerve (VII: red) and inferior olive (IO: orange).

Supplementary Image 2 **Retrograde transduction of spinally projecting neurons by HSV-hCMV-YTB**. Coronal section at the level of the RVLM showing YFP expression in spinally projecting neurons 7 days after vector injection. Reporter expression was predominantly ipsilateral to the injection side; most transduced neurons were found in the RVLM, RVMM, and ventral raphe nuclei.

Supplementary Image 3 **Spatial distribution of rat rostral ventrolateral medulla (RVLM) input neurons identified in the current study (magenta, plotted as distance from RVLM epicenter) compared to normalized nodal edge length distributions of reciprocal (blue) and non-reciprocal (black) nodes contained within the Allen Mouse Brain Connectivity Atlas**. Mouse connectivity data adapted with permission from Henriksen et al. (2016).

Video 1 **Positions of C1 (yellow) and non-C1 (green) starter neurons in Waxholm space**. Data shown first plotted on the Waxholm MRI volume, then in the Waxholm segmentation model, and finally (with monosynaptically connected input neurons, magenta), in the whole brain model.

Video 2 **Virtual flythrough showing the positions of monosynaptically connected input neurons colored according to cluster and projected onto the Waxholm MRI dataset**. The video starts at the caudal end of the Waxholm MRI dataset, presented in the coronal plane, and flies in the rostral direction. The audio channel denotes the number of input neurons per section.

Table S1 **RVLM input neurons sorted according to Waxholm-defined region**.

Table S2 **Key to cluster analysis and relevant literature**. Abbreviations: A1, A1 noradrenergic group; BötC, Bötzinger Complex; C3, C3 adrenergic group; CPA, caudal pressor area; KF, Kolliker-Fuse; LHA, lateral hypothalamic area; MCPA, medullocervical pressor area; MPB/LPB, medial & lateral parabrachial nucleus; mRaphe, midline Raphe; NTS, nucleus of the solitary tract; Pe, perifornical hypothalamus; PiCo, postinspiratory complex; Pr, nucleus prepositus; preBötC, pre-Bötzinger Complex; PVN, paraventricular nucleus; Ramb, nucleus retroambiguus; RVLM, rostral ventrolateral medulla; RVMM, rostral ventromedial medulla; rVRG/cVRG, rostral & caudal ventral respiratory group; SC, superior colliculus; SubLC, sub-coeruleus; VL/LPAG, ventrolateral & lateral periaqueductal gray; vLTF/dLTF, ventral & dorsal lateral tegmental field; ZI, zona incerta.

Data Sheet 1 **Waxholm co-ordinates of entire dataset, segregated by experiment number and cell type (C1 or non-C1 starter neurons and input neurons)**.

Data Sheet 2 **Waxholm co-ordinates and Kmeans analysis of input neurons, segregated by cluster number**.
