# Supplementary figures and images for: Mapping and Analysis of the Connectome of Sympathetic Premotor Neurons in the Rostral Ventrolateral Medulla of the Rat Using a Volumetric Brain Atlas
Source: Front Neural Circuits. 2017 Mar 1;11:9. doi: 10.3389/fncir.2017.00009 (PMC5331070; doi:10.3389/fncir.2017.00009)

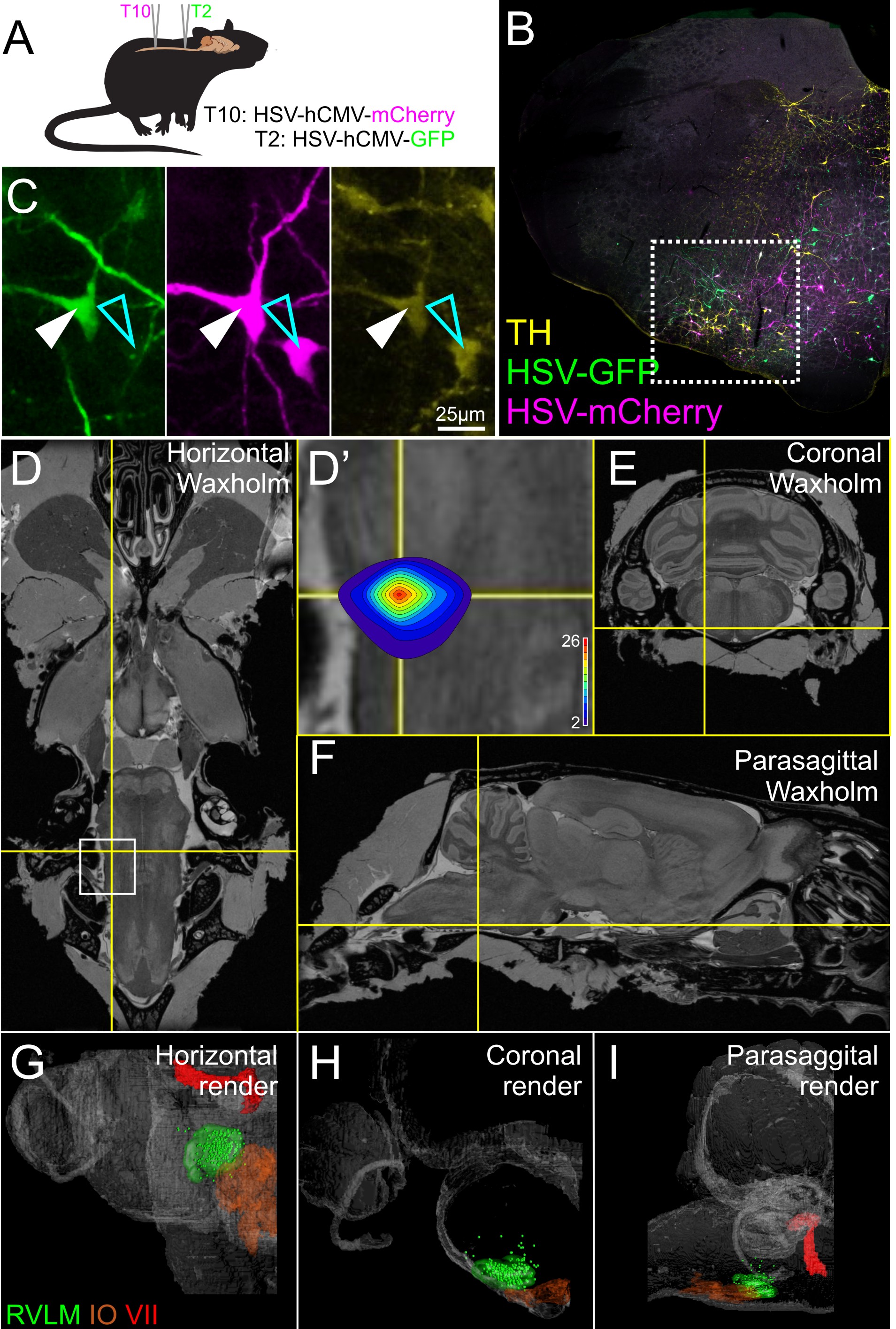

Supplement: Supplementary Image 1 — Segmentation of the RVLM in Waxholm space. (A) Experimental strategy: HSV vectors encoding GFP or mCherry were microinjected into the T2 or T10 spinal cord and (B) retrogradely transduced RVLM neurons (boxed area) were classified as C1 or non-C1; (C) shows examples of C1 neurons labeled from the T10 segment (open cyan arrowhead) and from both the T2 and T10 segments (closed white arrowhead). (D) Waxholm coordinates of 273 bulbospinal C1 neurons were plotted as 2d heat maps at each horizontal plane (detail in D'): the epicenter is indicated by crosshairs in the corresponding horizontal (D), coronal (E), and parasagittal (F) sections through the Waxholm MRI dataset. (G–I) Coordinates of individual bulbospinal C1 neurons (green spheres) and segmentation that enclosed 86% of neurons (green surface) plotted in 3d model of the Waxholm brain. Regional landmarks are the facial nerve (VII: red) and inferior olive (IO: orange). [file Image1.JPEG]

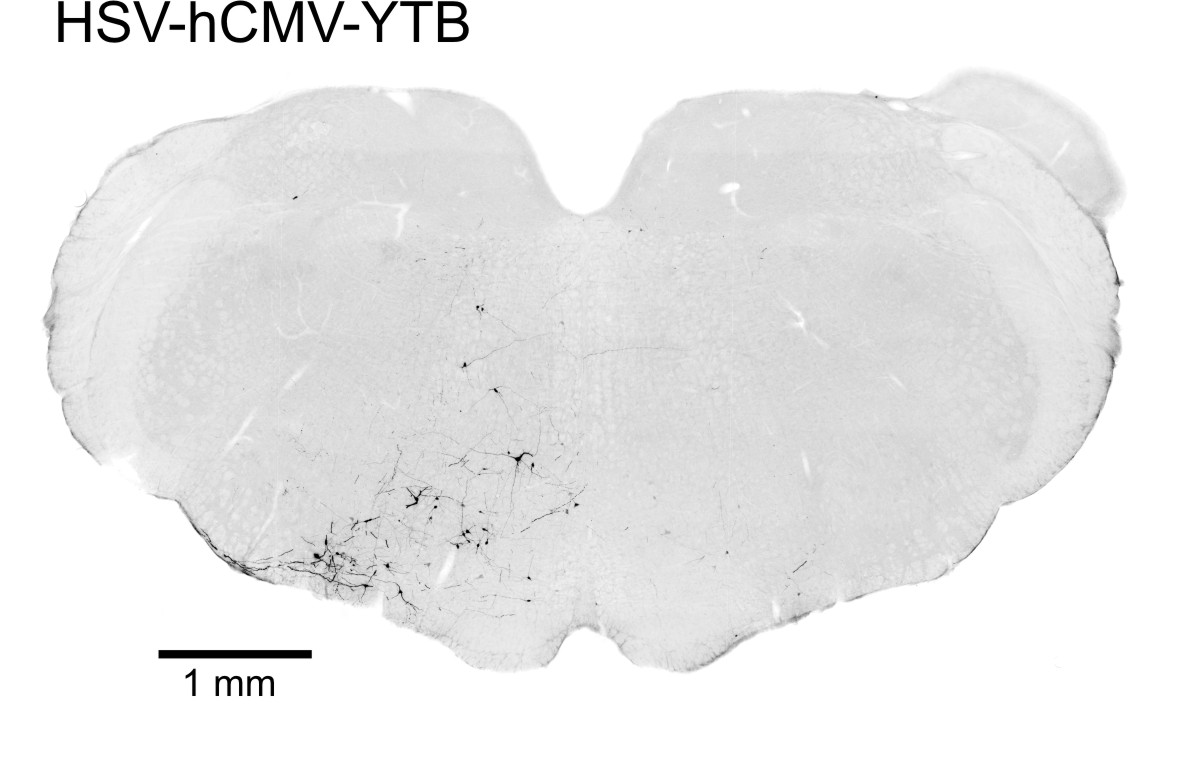

Supplement: Supplementary Image 2 — Retrograde transduction of spinally projecting neurons by HSV-hCMV-YTB. Coronal section at the level of the RVLM showing YFP expression in spinally projecting neurons 7 days after vector injection. Reporter expression was predominantly ipsilateral to the injection side; most transduced neurons were found in the RVLM, RVMM, and ventral raphe nuclei. [file Image2.JPEG]

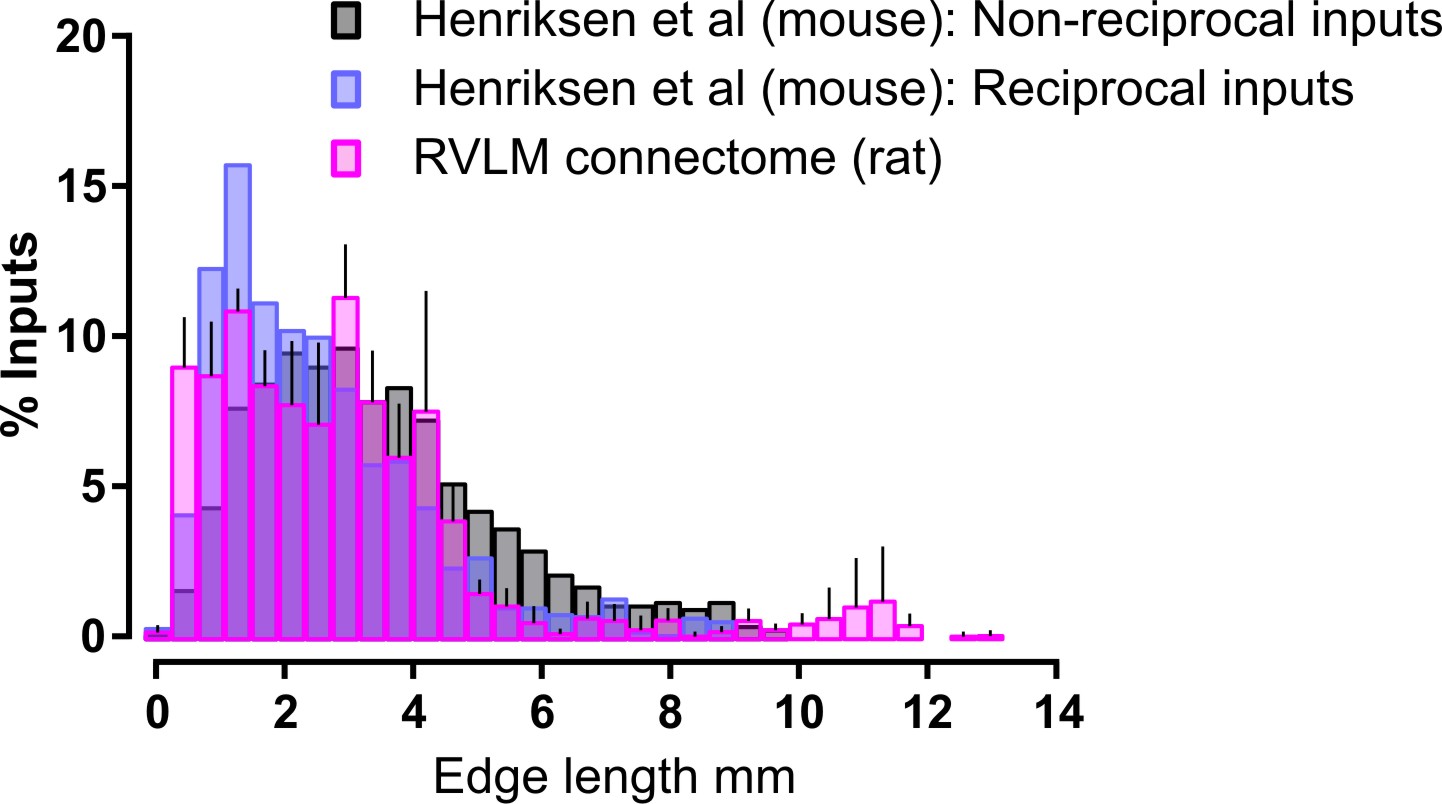

Supplement: Supplementary Image 3 — Spatial distribution of rat rostral ventrolateral medulla (RVLM) input neurons identified in the current study (magenta, plotted as distance from RVLM epicenter) compared to normalized nodal edge length distributions of reciprocal (blue) and non-reciprocal (black) nodes contained within the Allen Mouse Brain Connectivity Atlas. Mouse connectivity data adapted with permission from Henriksen et al. (2016). [file Image3.JPEG]
