# Supplementary material for: Mapping and Analysis of the Connectome of Sympathetic Premotor Neurons in the Rostral Ventrolateral Medulla of the Rat Using a Volumetric Brain Atlas
Source: Front Neural Circuits. 2017 Mar 1;11:9. doi: 10.3389/fncir.2017.00009 (PMC5331070; doi:10.3389/fncir.2017.00009)
Supplement: Table S1 — RVLM input neurons sorted according to Waxholm-defined region. [file Table1.DOCX]

| **Brain Region**/Subnucleus | # neurons | neurons/seed  % total, mean + SEM | Volume of region kvoxels | Input Density %neurons/seed/Mvoxel |
| --- | --- | --- | --- | --- |
| **Forebrain** | **31** | **3.07 ± 2.4** |  |  |
| Neocortex | 1 | 0.05 ± 0.05 | 10500 | 0±0 |
| Nucleus of the stria medullaris | 1 | 0.09 ± 0.1 | 1.6 | 55.9±64.5 |
| Thalamus | 25 | 2.56 ± 2.22 | 1390 | 1.8±1.6 |
| Basal forebrain region | 4 | 0.37 ± 0.23 | 1250 | 0.3±0.2 |
| **Midbrain** | **34** | **3.06 ± 1.00** |  |  |
| Hypothalamus | 12 | 1.21 ± 0.52 | 388 | 3.1±1.3 |
| Periaqueductal grey | 15 | 1.23 ± 0.35 | 269 | 4.6±1.3 |
| Superior colliculus | 5 | 0.43 ± 0.27 | 490 | 1.1±0.7 |
| Inferior colliculus | 1 | 0.09 ± 0.10 | 451 | 0.2±0.2 |
| Substantia Nigra | 1 | 0.09 ± 0.10 | 114 | 0.8±0.9 |
| **Cerebellum** |  |  |  |  |
| Cerebellum granular layer | 4 | 0.25 ± 0.17 | 2730 | 0.1±0.1 |
| **Brainstem** | **1159** | **91.95 ± 1.85** |  |  |
| Pyramidal tract/corticofugal pathway | 2 | 0.21 ± 0.14 | 461 | 0.4±0.3 |
| Periventricular grey | 40 | 2.44 ± 1.21 | 226 | 10.8±5.4 |
| Inferior olive | 4 | 0.34 ± 0.18 | 33 | 10.3±5.5 |
| Spinal trigeminal nucleus | 13 | 1.12 ± 0.37 | 252 | 4.5±1.5 |
| Spinal trigeminal tract | 8 | 0.65 ± 0.51 | 1551 | 0.4±0.3 |
| Facial nucleus/perifacial region | 22 | 2.27 ± 1.44 | 31.6 | 71.8±45.4 |
| Nucleus ambiguus | 14 | 0.97 ± 0.38 | 0.5 | 1935.4±755.2 |
| Bötzinger | 9 | 0.71 ± 0.11 | 3.1 | 232.3±35.9 |
| Pre-Bӧtzinger Complex | 16 | 1.13 ± 0.65 | 2.4 | 451.8±259.5 |
| RVLM | 176 | 14.10 ± 1.64 | 46 | 306.6±35.7 |
| Brainstem (not specified) | 855 | 68.02 ± 4.16 | 3343 | 20.3±1.2 |
| **Spinal Cord** | **25** | **1.67 ± 0.74** | **1590** | **1.1±0.5** |

Table S1: RVLM input neurons sorted according to Waxholm-defined region
