# Supplementary material for: Mapping and Analysis of the Connectome of Sympathetic Premotor Neurons in the Rostral Ventrolateral Medulla of the Rat Using a Volumetric Brain Atlas
Source: Front Neural Circuits. 2017 Mar 1;11:9. doi: 10.3389/fncir.2017.00009 (PMC5331070; doi:10.3389/fncir.2017.00009)
Supplement: Table S2 — Key to cluster analysis and relevant literature. Abbreviations: A1, A1 noradrenergic group; BötC, Bötzinger Complex; C3, C3 adrenergic group; CPA, caudal pressor area; KF, Kolliker-Fuse; LHA, lateral hypothalamic area; MCPA, medullocervical pressor area; MPB/LPB, medial & lateral parabrachial nucleus; mRaphe, midline Raphe; NTS, nucleus of the solitary tract; Pe, perifornical hypothalamus; PiCo, postinspiratory complex; Pr, nucleus prepositus; preBötC, pre-Bötzinger Complex; PVN, paraventricular nucleus; Ramb, nucleus retroambiguus; RVLM, rostral ventrolateral medulla; RVMM, rostral ventromedial medulla; rVRG/cVRG, rostral & caudal ventral respiratory group; SC, superior colliculus; SubLC, sub-coeruleus; VL/LPAG, ventrolateral & lateral periaqueductal gray; vLTF/dLTF, ventral & dorsal lateral tegmental field; ZI, zona incerta. [file Table2.docx]

| **Cluster #** | **Color in Figures** | **Centroid location (ML, RC, DV Waxholm space)** | **Sum of squares (square** | ***n* inputs** | **Structures enveloped** | **References describing projections to the RVLM** |
| --- | --- | --- | --- | --- | --- | --- |
| 1 | Red | 188.3, 385.9, 235.2 | 66197 | 44 | A5, SubLC, KF, MPB,LPB | (Dampney et al., 1982;Lovick, 1986;Dampney et al., 1987;Van Bockstaele et al., 1989;Hayakawa et al., 1999;Goodchild et al., 2001;Card et al., 2011) |
| 2 | Orange | 197.2, 310.4, 194.2 | 88306 | 294 | vLTF, BötC, C1/RVLM/PiCo | (Barman and Gebber, 1987;Gebber and Barman, 1988;Nicholas and Hancock, 1991;Bryant et al., 1993;Granata and Chang, 1994;Lipski et al., 1995;Gaytan et al., 1997;Sun et al., 1997;Madden et al., 1999;Card et al., 2006;Card et al., 2011;Agassandian et al., 2012;McMullan and Pilowsky, 2012;Turner et al., 2013) (Anderson et al., 2016) |
| 3 | Yellow | 216, 307.1, 234.4 | 63460 | 144 | dLTF | (Barman and Gebber, 1987;Gebber and Barman, 1988) |
| 4 | White (Movie 2)/Black (Figure 3) | 197.3, 276.3, 202.2 | 50340 | 140 | rVRG, C1/CVLM, preBötC | (Willette et al., 1984;Blessing, 1988;Ellenberger and Feldman, 1990;Agarwal and Calaresu, 1991;Gieroba et al., 1992;Li et al., 1992;Gaytan et al., 1997;Chan and Sawchenko, 1998;Tan et al., 2010) |
| 5 | Green | 288.7, 290.0, 205.3 | 81917 | 130 | (Contralateral) CPA, rVRG, C1/CVLM, preBötC, BötC, RVLM | (Willette et al., 1984;Lovick, 1986;Blessing, 1988;Gordon and McCann, 1988;Ellenberger and Feldman, 1990;Agarwal and Calaresu, 1991;Nicholas and Hancock, 1991;Gieroba et al., 1992;Li et al., 1992;Bryant et al., 1993;Granata and Chang, 1994;Possas et al., 1994;Lipski et al., 1995;Gaytan et al., 1997;Sun et al., 1997;Chan and Sawchenko, 1998;Madden et al., 1999;Sun and Panneton, 2002;2005;Card et al., 2006;Tan et al., 2010;Card et al., 2011;Agassandian et al., 2012;McMullan and Pilowsky, 2012;Turner et al., 2013) |
| 6 | Dark Green | 237.7, 247.4, 237.4 | 60796 | 76 | NTS | (Dampney et al., 1982;Ross et al., 1985;Lovick, 1986;Dampney et al., 1987;Aicher et al., 1996;Koshiya and Guyenet, 1996) |
| 7 | Cyan | 232.7, 335, 186.7 | 71074 | 115 | RVMM, mRaphe | (Lovick, 1986;Van Bockstaele et al., 1989;Zagon, 1995;Milner et al., 1996;Babic and Ciriello, 2004;Card et al., 2011) |
| 8 | Blue | 241, 460.1, 284.3 | 100117 | 38 | VLPAG, LPAG, SC | (Lovick, 1985;Carrive et al., 1988;Van Bockstaele et al., 1989;Card et al., 2011;Stornetta et al., 2015) |
| 9 | Purple | 231.6, 332.0, 243.6 | 59628 | 136 | Pr/C3 area | (Lovick, 1986;Sevigny et al., 2012) |
| 10 | Magenta | 204.8, 231.2, 205.2 | 53172 | 77 | A1, cVRG/Ramb, MCPA/CPA | (Granata et al., 1986;Dampney et al., 1987;Gordon and McCann, 1988;Possas et al., 1994;Chan and Sawchenko, 1998;Sun and Panneton, 2002;2005) |
| 11 | Brown | 280.5, 357.3, 231.1 | 84956 | 55 | (Contralateral) A5, SubLC, KF, MPB, LPB | (Dampney et al., 1982;Lovick, 1986;Dampney et al., 1987;Hayakawa et al., 1999;Goodchild et al., 2001;Card et al., 2011) |
| 12 | Pink | 232.3, 569.1, 224.8 | 103724 | 49 | Pe, LHA, PVN, ZI | (Lovick, 1985;Dampney et al., 1987;Van Bockstaele et al., 1989;Hardy, 2001;Card et al., 2011) |

Table S2: Key to cluster analysis and relevant literature. Abbreviations: A1: A1 noradrenergic group, BötC: Bötzinger Complex, C3: C3 adrenergic group, CPA: caudal pressor area, KF: Kolliker-Fuse, LHA: lateral hypothalamic area, MCPA: medullocervical pressor area, MPB/LPB: medial & lateral parabrachial nucleus, mRaphe: midline Raphe, NTS: nucleus of the solitary tract, Pe: perifornical hypothalamus, PiCo: postinspiratory complex, Pr: nucleus prepositus, preBötC: pre-Bötzinger Complex, PVN: paraventricular nucleus, Ramb: nucleus retroambiguus, RVLM: rostral ventrolateral medulla, RVMM: rostral ventromedial medulla, rVRG/cVRG: rostral & caudal ventral respiratory group, SC: superior colliculus, SubLC: sub-coeruleus, VL/LPAG: ventrolateral & lateral periaqueductal grey, vLTF/dLTF: ventral & dorsal lateral tegmental field, ZI: zona incerta.

# Supplementary References

Agarwal, S.K., and Calaresu, F.R. (1991). Monosynaptic connection from caudal to rostral ventrolateral medulla in the baroreceptor reflex pathway. *Brain Res* 555**,** 70-74.

Agassandian, K., Shan, Z., Raizada, M., Sved, A.F., and Card, J.P. (2012). C1 catecholamine neurons form local circuit synaptic connections within the rostroventrolateral medulla of rat. *Neuroscience* 227**,** 247-259.

Aicher, S.A., Saravay, R.H., Cravo, S., Jeske, I., Morrison, S.F., Reis, D.J., and Milner, T.A. (1996). Monosynaptic projections from the nucleus tractus solitarii to C1 adrenergic neurons in the rostral ventrolateral medulla: comparison with input from the caudal ventrolateral medulla. *J Comp Neurol* 373**,** 62-75.

Anderson, T.M., Garcia, A.J., Baertsch, N.A., Pollak, J., Bloom, J.C., Wei, A.D., Rai, K.G., and Ramirez, J.-M. (2016). A novel excitatory network for the control of breathing. *Nature* 536**,** 76-80.

Babic, T., and Ciriello, J. (2004). Medullary and spinal cord projections from cardiovascular responsive sites in the rostral ventromedial medulla. *J Comp Neurol* 469**,** 391-412.

Barman, S.M., and Gebber, G.L. (1987). Lateral tegmental field neurons of cat medulla: a source of basal activity of ventrolateral medullospinal sympathoexcitatory neurons. *J Neurophysiol* 57**,** 1410-1424.

Blessing, W.W. (1988). Depressor neurons in rabbit caudal medulla act via GABA receptors in rostral medulla. *Am J Physiol* 254**,** H686-692.

Bryant, T.H., Yoshida, S., De Castro, D., and Lipski, J. (1993). Expiratory neurons of the Botzinger Complex in the rat: a morphological study following intracellular labeling with biocytin. *J Comp Neurol* 335**,** 267-282.

Card, J.P., Kobiler, O., Mccambridge, J., Ebdlahad, S., Shan, Z., Raizada, M.K., Sved, A.F., and Enquist, L.W. (2011). Microdissection of neural networks by conditional reporter expression from a Brainbow herpesvirus. *Proceedings of the National Academy of Sciences of the United States of America* 108**,** 3377-3382.

Card, J.P., Sved, J.C., Craig, B., Raizada, M., Vazquez, J., and Sved, A.F. (2006). Efferent projections of rat rostroventrolateral medulla C1 catecholamine neurons: Implications for the central control of cardiovascular regulation. *The Journal of Comparative Neurology* 499**,** 840-859.

Carrive, P., Bandler, R., and Dampney, R.A. (1988). Anatomical evidence that hypertension associated with the defence reaction in the cat is mediated by a direct projection from a restricted portion of the midbrain periaqueductal grey to the subretrofacial nucleus of the medulla. *Brain Res* 460**,** 339-345.

Chan, R.K., and Sawchenko, P.E. (1998). Organization and transmitter specificity of medullary neurons activated by sustained hypertension: implications for understanding baroreceptor reflex circuitry. *J Neurosci* 18**,** 371-387.

Dampney, R.A., Czachurski, J., Dembowsky, K., Goodchild, A.K., and Seller, H. (1987). Afferent connections and spinal projections of the pressor region in the rostral ventrolateral medulla of the cat. *J Auton Nerv Syst* 20**,** 73-86.

Dampney, R.A., Goodchild, A.K., Robertson, L.G., and Montgomery, W. (1982). Role of ventrolateral medulla in vasomotor regulation: a correlative anatomical and physiological study. *Brain Res* 249**,** 223-235.

Ellenberger, H.H., and Feldman, J.L. (1990). Brainstem connections of the rostral ventral respiratory group of the rat. *Brain Res* 513**,** 35-42.

Gaytan, S.P., Calero, F., Nunez-Abades, P.A., Morillo, A.M., and Pasaro, R. (1997). Pontomedullary efferent projections of the ventral respiratory neuronal subsets of the rat. *Brain Res Bull* 42**,** 323-334.

Gebber, G.L., and Barman, S.M. (1988). Studies on the origin and generation of sympathetic nerve activity. *Clin.Exp.Hypertens.A* 10 Suppl 1:33-44.**,** 33-44.

Gieroba, Z.J., Li, Y.W., and Blessing, W.W. (1992). Characteristics of caudal ventrolateral medullary neurons antidromically activated from rostral ventrolateral medulla in the rabbit. *Brain Res* 582**,** 196-207.

Goodchild, A.K., Phillips, J.K., Lipski, J., and Pilowsky, P.M. (2001). Differential expression of catecholamine synthetic enzymes in the caudal ventral pons. *Journal of Comparative Neurology* 438**,** 457-467.

Gordon, F.J., and Mccann, L.A. (1988). Pressor responses evoked by microinjections of L-glutamate into the caudal ventrolateral medulla of the rat. *Brain Res* 457**,** 251-258.

Granata, A.R., and Chang, H.T. (1994). Relationship of calbindin D-28k with afferent neurons to the rostral ventrolateral medulla in the rat. *Brain Res* 645**,** 265-277.

Granata, A.R., Numao, Y., Kumada, M., and Reis, D.J. (1986). A1 noradrenergic neurons tonically inhibit sympathoexcitatory neurons of C1 area in rat brainstem. *Brain Res* 377**,** 127-146.

Hardy, S.G. (2001). Hypothalamic projections to cardiovascular centers of the medulla. *Brain Res* 894**,** 233-240.

Hayakawa, T., Zheng, J.Q., and Seki, M. (1999). Direct parabrachial nuclear projections to the pharyngeal motoneurons in the rat: an anterograde and retrograde double-labeling study. *Brain Res* 816**,** 364-374.

Koshiya, N., and Guyenet, P.G. (1996). NTS neurons with carotid chemoreceptor inputs arborize in the rostral ventrolateral medulla. *American Journal of Physiology-Regulatory Integrative and Comparative Physiology* 39**,** R1273-R1278.

Li, Y.W., Wesselingh, S.L., and Blessing, W.W. (1992). Projections from rabbit caudal medulla to C1 and A5 sympathetic premotor neurons, demonstrated with phaseolus leucoagglutinin and herpes simplex virus. *J Comp Neurol* 317**,** 379-395.

Lipski, J., Kanjhan, R., Kruszewska, B., and Smith, M. (1995). Barosensitive neurons in the rostral ventrolateral medulla of the rat in vivo: morphological properties and relationship to C1 adrenergic neurons. *Neuroscience* 69**,** 601-618.

Lovick, T.A. (1985). Projections from the diencephalon and mesencephalon to nucleus paragigantocellularis lateralis in the cat. *Neuroscience* 14**,** 853-861.

Lovick, T.A. (1986). Projections from brainstem nuclei to the nucleus paragigantocellularis lateralis in the cat. *J Auton Nerv Syst* 16**,** 1-11.

Madden, C.J., Ito, S., Rinaman, L., Wiley, R.G., and Sved, A.F. (1999). Lesions of the C1 catecholaminergic neurons of the ventrolateral medulla in rats using anti-DbetaH-saporin. *Am J Physiol* 277**,** R1063-1075.

Mcmullan, S., and Pilowsky, P.M. (2012). Sympathetic premotor neurones project to and are influenced by neurones in the contralateral rostral ventrolateral medulla of the rat in vivo. *Brain Res* 1439**,** 34-43.

Milner, T.A., Reis, D.J., and Giuliano, R. (1996). Afferent sources of substance P in the C1 area of the rat rostral ventrolateral medulla. *Neurosci Lett* 205**,** 37-40.

Nicholas, A.P., and Hancock, M.B. (1991). Projections from the rostral ventrolateral medulla to brainstem monoamine neurons in the rat. *Neurosci Lett* 122**,** 91-95.

Possas, O.S., Campos, R.R., Jr., Cravo, S.L., Lopes, O.U., and Guertzenstein, P.G. (1994). A fall in arterial blood pressure produced by inhibition of the caudalmost ventrolateral medulla: the caudal pressor area. *J Auton Nerv Syst* 49**,** 235-245.

Ross, C.A., Ruggiero, D.A., and Reis, D.J. (1985). Projections from the nucleus tractus solitarii to the rostral ventrolateral medulla. *J Comp Neurol* 242**,** 511-534.

Sevigny, C.P., Bassi, J., Williams, D.A., Anderson, C.R., Thomas, W.G., and Allen, A.M. (2012). Efferent projections of C3 adrenergic neurons in the rat central nervous system. *J Comp Neurol* 520**,** 2352-2368.

Stornetta, R.L., Inglis, M.A., Viar, K.E., and Guyenet, P.G. (2015). Afferent and efferent connections of C1 cells with spinal cord or hypothalamic projections in mice. *Brain Struct Funct*.

Sun, Q.J., Minson, J., Llewellyn-Smith, I.J., Arnolda, L., Chalmers, J., and Pilowsky, P. (1997). Botzinger neurons project towards bulbospinal neurons in the rostral ventrolateral medulla of the rat. *Journal of Comparative Neurology* 388**,** 23-31.

Sun, W., and Panneton, W.M. (2002). The caudal pressor area of the rat: its precise location and projections to the ventrolateral medulla. *Am J Physiol Regul Integr Comp Physiol* 283**,** R768-778.

Sun, W., and Panneton, W.M. (2005). Defining projections from the caudal pressor area of the caudal ventrolateral medulla. *J Comp Neurol* 482**,** 273-293.

Tan, W., Pagliardini, S., Yang, P., Janczewski, W.A., and Feldman, J.L. (2010). Projections of preBotzinger complex neurons in adult rats. *J Comp Neurol* 518**,** 1862-1878.

Turner, A., Kumar, N., Farnham, M., Lung, M., Pilowsky, P., and Mcmullan, S. (2013). Rostroventrolateral medulla neurons with commissural projections provide input to sympathetic premotor neurons: anatomical and functional evidence. *Eur J Neurosci* 38**,** 2504-2515.

Van Bockstaele, E.J., Pieribone, V.A., and Aston-Jones, G. (1989). Diverse afferents converge on the nucleus paragigantocellularis in the rat ventrolateral medulla: retrograde and anterograde tracing studies. *J Comp Neurol* 290**,** 561-584.

Willette, R.N., Punnen, S., Krieger, A.J., and Sapru, H.N. (1984). Interdependence of rostral and caudal ventrolateral medullary areas in the control of blood pressure. *Brain Research* 321**,** 169-174.

Zagon, A. (1995). Internal connections in the rostral ventromedial medulla of the rat. *J Auton Nerv Syst* 53**,** 43-56.
